# Increased Expression of Serglycin in Specific Carcinomas and Aggressive Cancer Cell Lines

**DOI:** 10.1155/2015/690721

**Published:** 2015-10-25

**Authors:** Angeliki Korpetinou, Dionysios J. Papachristou, Angeliki Lampropoulou, Panagiotis Bouris, Vassiliki T. Labropoulou, Argyrios Noulas, Nikos K. Karamanos, Achilleas D. Theocharis

**Affiliations:** ^1^Biochemistry, Biochemical Analysis & Matrix Pathobiology Research Group, Laboratory of Biochemistry, Department of Chemistry, University of Patras, 26500 Patras, Greece; ^2^Department of Anatomy/Histology/Embryology, Unit of Bone and Soft Tissue Studies, School of Medicine, University of Patras, 26500 Patras, Greece; ^3^Hematology Division, Department of Internal Medicine, University Hospital of Patras, 26500 Patras, Greece; ^4^School of Health Professions, Department of Medical Laboratories, Technological Educational Institute of Larissa, 41110 Larissa, Greece

## Abstract

In the present pilot study, we examined the presence of serglycin in lung, breast, prostate, and colon cancer and evaluated its expression in cell lines and tissues. We found that serglycin was expressed and constitutively secreted in culture medium in high levels in more aggressive cancer cells. It is worth noticing that aggressive cancer cells that harbor KRAS or EGFR mutations secreted serglycin constitutively in elevated levels. Furthermore, we detected the transcription of an alternative splice variant of serglycin lacking exon 2 in specific cell lines. In a limited number of tissue samples analyzed, serglycin was detected in normal epithelium but was also expressed in higher levels in advanced grade tumors as shown by immunohistochemistry. Serglycin staining was diffuse, granular, and mainly cytoplasmic. In some cancer cells serglycin also exhibited membrane and/or nuclear immunolocalization. Interestingly, the stromal cells of the reactive tumor stroma were positive for serglycin, suggesting an enhanced biosynthesis for this proteoglycan in activated tumor microenvironment. Our study investigated for first time the distribution of serglycin in normal epithelial and cancerous lesions in most common cancer types. The elevated levels of serglycin in aggressive cancer and stromal cells may suggest a key role for serglycin in disease progression.

## 1. Introduction

Proteoglycans are composed of a specific core protein substituted with one or more covalently linked glycosaminoglycan chains. Proteoglycans are either secreted in the extracellular matrix or are located at the cell membrane and intracellularly [1]. They participate in the organization of extracellular matrix but also regulate cell phenotype and properties in tissues [2]. Proteoglycans are synthesized by tumor and stromal cells and their biosynthesis is often dysregulated in malignancies, providing a favorable microenvironment for disease progression [2].

Serglycin is the only characterized intracellular proteoglycan till now and has been initially regarded as “hematopoietic” proteoglycan, being detected mainly in the secretory granules of hematopoietic cells [3, 4]. Numerous studies have shown that serglycin is constitutively secreted by tumor cells and in some cases is also located at the tumor cell membrane, although it does not hold a transmembrane domain [5–7]. Serglycin is highly expressed and secreted by tumor cells themselves and its overexpression is associated with tumor cell aggressiveness and poor disease outcome [8–10]. It is the major proteoglycan secreted by multiple myeloma cells affecting bone mineralization [7] growth of myeloma cell* in vivo* and secretion of hepatocyte growth factor (HGF) [5]. Cell surface associated serglycin in myeloma cells is involved in cell adhesion to collagen type I and stromal cells [5, 11]. The adhesion of myeloma cells to collagen type I enhances the biosynthesis of matrix metalloproteinases (MMPs) [11]. Furthermore, secreted and cell surface associated serglycin is capable of inhibiting the classical and lectin pathways of complement via its chondroitin sulfate (CS) chains, thus protecting tumor cells from complement system attack [9, 12].

Few recent studies have demonstrated the overexpression of serglycin by aggressive cancer cells in tumors [8–10]. The upregulated biosynthesis and secretion of glycanated serglycin by cancer cells promote their growth, migration, and invasion and are correlated with poor prognosis [8–10]. Since little is known on the expression of serglycin in solid tumors, we went on to study the expression and distribution of serglycin in cancer cell lines and malignant tissues. In our pilot study, we show that serglycin is differentially expressed and secreted by breast, prostate, lung, and colon cancer cell lines. We identify the transcript variant of serglycin missing exon 2 in several of these cell lines. Our findings that serglycin is markedly synthesized by cancer and stromal cells in malignant tissues may propose a role for serglycin in cancer progression.

## 2. Materials and Methods

### 2.1. Antibodies, Enzymes, and Purified Proteins

Goat anti-rabbit horseradish peroxidase- (HRP-) conjugated secondary antibody was from Sigma-Aldrich. Rabbit polyclonal antibody against serglycin was prepared as previously described [7]. Chondroitinase ABC was purchased from Seikagaku. Serglycin isolated from culture medium of multiple myeloma cell lines was used as standard [7].

### 2.2. Cell Culture

All cell lines were purchased from the American Type Culture Collection (ATCC). MDA-MB-468, DLD-1, HT-29, A549, NCI-H23, NCI-H358, NCI-H661, HCC827, and PC-3 cells were cultured in RPMI 1640 medium (Biochrom) with 2 mM L-glutamine supplemented with 10 mM HEPES, 1 mM sodium pyruvate, 4.5 g/L glucose (except for PC-3 cells) or 0.1 g/L (PC-3 cells), 1.5 g/L sodium bicarbonate, and 10% fetal bovine serum as recommended by ATCC. MDA-MB-468 cells were also supplemented with 10 *μ*g/mL human insulin (Sigma-Aldrich). MDA-MB-231, MCF-7, and CACO-2 cells were cultured in Eagle's minimum essential medium with Earle's BSS and 2 mM L-glutamine (EMEM, Biochrom) and supplemented with 1 mM sodium pyruvate, 0.1 mM nonessential amino acids, 1.5 g/L sodium bicarbonate, 10 *μ*g/mL human insulin (MCF-7 cells), and 10% fetal bovine serum (MDA-MB-231 and MCF-7 cells) or 20% fetal bovine serum (CACO-2 cells) as recommended by ATCC. For each cell line, 1% Pen/Strep (10000 units/mL penicillin and 10000 units/mL streptomycin, Biochrom) was used. Cells were cultured at 37°C in 5% CO_2_.

### 2.3. Quantification of Serglycin Concentration in Culture Medium Supernatants

1 × 10^6^ cells were plated in 10 cm dish and cultured at normal culture conditions. After 18 h, cells were starved in serum-free medium for 48 h, when cultures reached 80% confluency and culture supernatants were collected. They were centrifuged at 3000 rpm for 5 min and were concentrated with Vivaspin 6 ultrafiltration devices (Sartorius Biotech). Protein concentration was measured by Coomassie Plus-Bradford Assay Kit (Thermo Scientific) and equal amounts of protein for every sample were treated with 0.02 units of chondroitinase ABC in 50 mM Tris-HCl pH 7.5 at 37°C for 2 h. Then, samples were reduced with *β*-mercaptoethanol in Laemmli sample buffer and were separated by SDS-PAGE electrophoresis. The proteins were transferred to Immobilon-P PVDF membranes (Millipore) and the membranes were blocked in 5% nonfat dry milk in PBS-0.1% Tween-20 for 2 h. Then, membranes were incubated with 0.55 *μ*g/mL rabbit polyclonal anti-serglycin overnight at 4°C, washed three times with PBS-0.1% Tween-20, and then incubated for 1 h at room temperature with peroxidase-conjugated secondary goat anti-rabbit antibody. The immunoreactive proteins were detected by using the chemiluminescence horseradish peroxidase Pierce ECL western blotting substrate, according to the manufacturer's instructions. Serglycin content was analyzed through western blotting using increasing amounts of standard serglycin to create a standard curve each time [9]. Both culture supernatant and standard serglycin were treated with 0.02 units of chondroitinase ABC as above. The quantification of protein band density was performed using Scion Image software.

### 2.4. RNA Isolation and Real Time qPCR Analysis

1 × 10^6^ cells were plated in 10 cm dishes and cultured at normal culture conditions. After 18 h, cells were starved in serum-free culture medium for 48 h, when cultures reached 80% confluency. Total RNA was isolated from cells using NucleoSpin RNA II Kit (Macherey-Nagel, Duren, Germany). The amount of isolated RNA was quantified by measuring its absorbance at 260 nm and the integrity of RNA was confirmed by electrophoresis on agarose gel stained with GelRed nucleic acid gel stain (Biotium). Total RNA was reverse transcribed using the PrimeScript 1st strand cDNA synthesis kit perfect real time (Takara Bio Inc., Japan) and KAPA Taq ReadyMix DNA Polymerase (KAPA BIOSYSTEMS). Real time PCR analysis was performed in 20 *μ*L reaction mixture, according to the manufacturer's instructions using gene-specific primers (serglycin forward: 5′-GTTGGCGTGCAGCTGGGAGA-3′ and serglycin reverse: 5′-GGCTCTCCGCGTAGGATAACCTTG-3′, GAPDH forward: 5′-AGGCTGTTGTCATACTTCTCAT-3′ and GAPDH reverse: 5′-GGAGTCCACTGGCGTCTT-3′). The amplification was performed using Rotor Gene Q (Qiagen, USA). All reactions were performed in triplicate and a standard curve was always included for each pair of primers for assay validation. In addition, a melting curve analysis was always performed for detecting the SYBR Green-based objective amplicon. To provide quantification, the point of product accumulation in the early logarithmic phase of the amplification plot was defined by assigning a fluorescence threshold above the background, defined as the threshold cycle (Ct) number. Relative expression was calculated by the ΔΔCt method. Τhe Ct of any gene of interest was normalized to the Ct of the normalizer (GAPDH).

### 2.5. DNA Fragment Identification

In order to isolate and sequence PCR products for serglycin gene expression within the cell lines reverse transcription-polymerase chain reaction (RT-PCR) was performed with DyNAzyme II DNA Polymerase kit (Finnzymes). Serglycin and *β*-actin transcript levels were detected using gene-specific primers (serglycin forward: 5′-AATGCAGTCGGCTTGTCCTG-3′ and serglycin reverse: 5′-TGGTGTCAAGGTGGGAAAAT-3′ resulting in amplicon size of 483 bp for full-length serglycin and 336 bp for serglycin transcript variant lacking exon 2, *β*-actin forward: 5′-GTGGGGCGCCCCAGGCACCA-3′ and *β*-actin reverse: 5′-CTCCTTAATGTCACGCACGATTTC-3′ resulting in amplicon size of 539 bp). For PCR reaction, samples containing 125 ng of cDNA were amplified in a total volume of 50 *μ*L [10 mM Tris-HCl pH 8.8, 50 mM KCl, 1.5 mM MgCl_2_, 0.1% Triton X-100, containing dNTP mix (each at 0.2 mM), both downstream and upstream primers (each at 200 nM), and 1 unit of DyNAzyme II DNA polymerase]. The PCR amplification was carried out as follows: 95°C for 20 sec, annealing temperature 51°C for 20 sec, and 72°C for 30 sec (MiniCycler, MJ Research). Equal volumes of the PCR products were electrophoresed on 1% agarose gel stained with GelRed nucleic acid gel stain (Biotium). For sequencing experiments, PCR products for serglycin were isolated from MDA-MB-231 and A549 cancer cells. The bands correspond to full-length serglycin and serglycin transcript variant lacking exon 2 was cut from the agarose gel and the DNA fragments were extracted and purified using Nucleospin Extract II kit (Macherey-Nagel) according to the manufacturer's instructions. DNA fragments were sequenced by automated sequencing in the institutional facility at the Karolinska Institute (Stockholm, Sweden).

### 2.6. Tissue Samples and Immunohistochemistry

In the present study, we used a tissue microarray (TMA) platform that contained 10 cases of each of the following 4 types of carcinomas: colon, breast, lung, and prostate (TP483 for colon, breast, prostate, and lung cancer, US Biomax Inc.). Clinicopathological data, including gender, age, tumor grade, and TNM stage, were available. Following deparaffinization in xylene and rehydration in graded ethanol, conventional immunohistochemistry was performed on the TMA slide as described previously [9], with the use of rabbit anti-serglycin antibody (1.38 *µ*g/mL). Specific binding was detected with the Dako REAL EnVision detection system (peroxidase/DAB+, rabbit/mouse) and visualized with diaminobenzidine. Myeloma section was used as a positive control. In negative control slides, the primary antibody was substituted with 1% TBS. The sections were counterstained with hematoxylin and serglycin staining was scored as follows: 0, no staining; 1+, weak; 2+, moderate; 3+, strong staining.

## 3. Results

### 3.1. Serglycin Is Differentially Expressed by Cancer Cells

**Figure 1:**
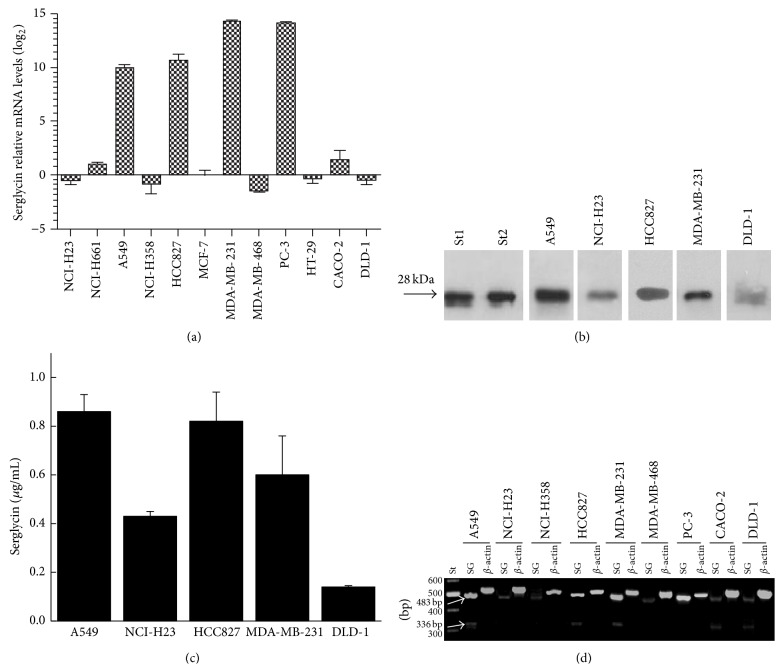
(a) Expression of serglycin across twelve cancer cell lines of lung (NCI-H23, NCI-H661, A549, and NCI-H358), breast (MCF-7, MDA-MB-231, and MDA-MB-468), prostate (PC-3), and colon (HT-29, CACO-2, and DLD-1). Results are mean of three separate experiments performed in triplicate ± S.D. (b) Equal amounts of protein from concentrated cell culture supernatants of cancer cell lines were treated with chondroitinase ABC and subjected to western blot analysis for serglycin using chondroitinase ABC digested standard serglycin (st1 and st2) as positive control. (c) Quantification of serglycin secreted in cell culture supernatants. Results are mean of three separate experiments performed in triplicate ± S.D. (d) Detection of serglycin gene transcripts by RT-PCR. The PCR products were analyzed on 1% agarose gels stained with GelRed.

Total RNA from A549, NCI-H23, NCI-H358, NCI-H661, and HCC827 lung cancer cells; MDA-MB-231, MDA-MB-468, and MCF-7 breast cancer cells; PC-3 prostate cancer cells; and CACO-2, DLD-1, and HT-29 colon cancer cells cultured in serum-free medium was reverse transcribed and amplified using specific primers for serglycin (Figure 1(a)). It was found that the expression of serglycin differs among cell lines and is likely aggressive-specific (Figure 1(a)). We have previously investigated serglycin expression in breast cancer cell lines MDA-MB-231, MDA-MB-468, and MCF-7 [9] of different aggressiveness [13, 14] by reverse transcription PCR. We went on to analyze the expression of serglycin in these breast cancer cell lines by qPCR and compare it to serglycin expression in cancer cell lines of different origin and tumorigenicity. In agreement with data presented in our previous study [9], serglycin was expressed in minute levels in low aggressive MDA-MB-468 and MCF-7 cells, whereas it was expressed in elevated levels in high aggressive MDA-MD-231 breast cancer cells, which are *KRAS* ^38G→A^ and *BRAF* ^1391G→T^ mutant (Figure 1(a)) [13, 14]. In accordance with these data, highly tumorigenic *KRAS* ^12G→S^ mutation activated A549 [15, 16] and *HER*1/*EGFR* ^Δ*E*746–750^ kinase domain mutation activated HCC827 lung cancer cells [17] expressed much higher levels of serglycin as compared to less tumorigenic *KRAS* ^12G→C^ mutant NCI-H358 and NCI-H23 as well as nonmutated H661 lung cancer cells [15, 16, 18] (Figure 1(a)). Serglycin expression was also significantly elevated in highly aggressive and tumorigenic [19] PC-3 prostate cancer cells (Figure 1(a)). The expression of serglycin was found to be relatively low in colon cancer cell lines and surprisingly was higher in low aggressive CACO-2 cells as compared to more aggressive HT-29 (mutant *BRAF* ^600V→T^) and DLD-1 (mutant *KRAS* ^13G→D^) colon cancer cells [20, 21] (Figure 1(a)).

In order to evaluate whether serglycin is secreted in the culture medium of cancer cells, they were cultured in serum-free medium and supernatants were collected and concentrated. Equal amounts of protein for every sample were digested with chondroitinase ABC and analyzed by western blot (Figure 1(b)). Serglycin core protein was detected only in the culture medium of aggressive cell lines such as A549, NCI-H23, and HCC827 lung cancer cells and DLD-1 colon cancer cells as well as MDA-MB-231 breast cancer cells as previously shown [9], which harbor KRAS or HER1/EGFR mutations (Figure 1(b)). It is worth noticing that aggressive NCI-H23 and DLD-1 cancer cells expressing low mRNA levels for serglycin secreted significant amounts of this proteoglycan. In contrast, aggressive PC-3 cells that highly express mRNA for serglycin did not secrete detectable amounts of this proteoglycan. We did not analyze cell culture supernatants from NCI-H358 lung cancer cell which are also KRAS mutant. We went on to quantify the secreted serglycin by western blot analysis creating a standard curve using various amounts of standard serglycin. Using standard curves, we simultaneously analyzed serglycin present in the culture medium of cancer cells and we found that the concentration of serglycin was as follows: A549, 0.86 ± 0.07 *μ*g/mL; NCI-H23, 0.43 ± 0.02 *μ*g/mL; HCC827, 0.82 ± 0.12 *μ*g/mL; MDA-MB-231, 0.6 ± 0.16 *μ*g/mL; and DLD-1, 0.14 ± 0.05 *μ*g/mL (Figure 1(c)).

### 3.2. Identification of Alternative Splicing of Serglycin in Cancer Cells

RT-PCR followed by agarose electrophoresis revealed the presence of two bands of RT-PCR products in A549 and HCC827 lung cancer cells; MBA-MB-231 breast cancer cells; and CACO-2 and DLD-1 colon cancer cells (Figure 1(d)). The upper band was the expected 483 bp fragment that represents the full-length sequence between exons 1 and 3 (bases 106–589) of human serglycin gene (Figure 1(d)). The lower band of 336 bp might correspond to an exon 2 deletion (Figure 1(d)). To identify both bands, RNA isolated from MDA-MB-231 and A549 cancer cells was subjected to RT-PCR followed by agarose electrophoresis. Both bands were extracted from the gel, purified, and sequenced. The sequencing verified the 483 bp fragment as the product of full-length sequence between exons 1 and 3 (bases 106–589) of human serglycin gene and showed that the reduced size of the 336 bp band was due to complete absence of exon 2 of serglycin gene. The partial sequence around this alternative splice site was tggaatcctcagttcaaGAagacgagaatccaggac, where the capital letters show the junction between exon 1 and exon 3 of serglycin gene. The loss of exon 2 (49 amino acids) has been also demonstrated in human neutrophils [22]; however, alternative splicing of serglycin in tumor cells has not been previously reported.

### 3.3. Distribution of Serglycin in Normal and Malignant Tissues

#### 3.3.1. Colon Cancer

**Figure 2:**
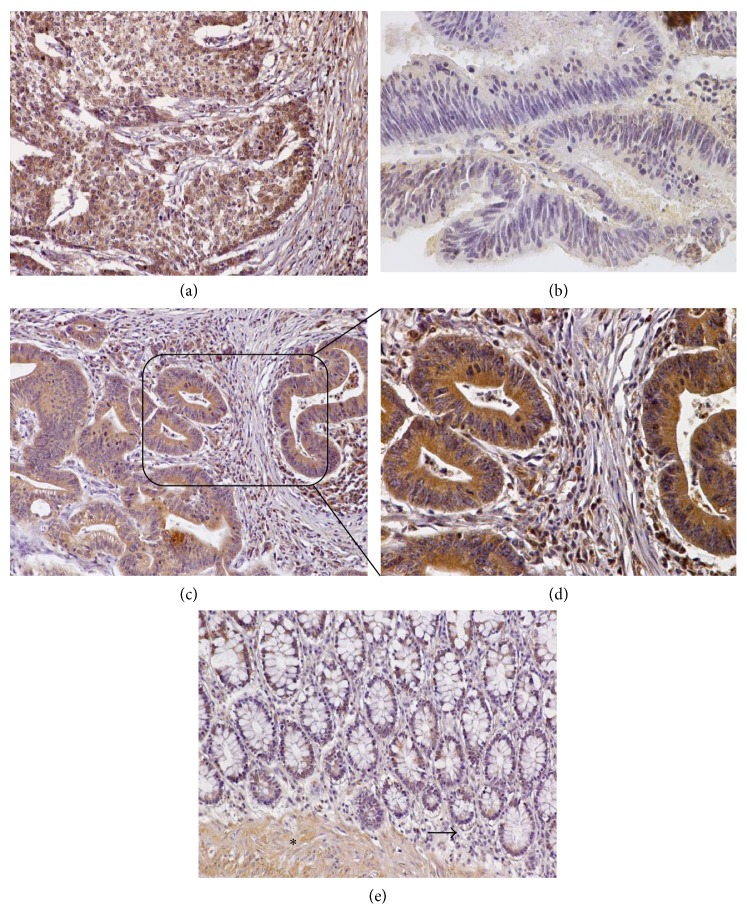
(a) Intense, diffuse cytoplasmic serglycin staining in a poorly differentiated colon adenocarcinoma. (b) Weak immunopositivity for serglycin in a well-differentiated adenocarcinoma, arising from an adenoma. (c) Moderately differentiated colon adenocarcinoma displaying strong immunoreactivity for serglycin. (d) Lager magnification showing that plasma cells, lymphocytes, and stromal cells of the invasive front are positive for serglycin. (e) Normal colon glands exhibit moderate cytoplasmic levels for serglycin. Note that muscularis mucosa (asterisk) is positive for serglycin, whereas staining is weak in plasma cells, lymphocytes, and stromal cells of the lamina propria (arrow). (a, b, c, e) Original magnification 20x; (d) original magnification 40x.

In the present tissue microarray, there were 10 colon carcinomas of different grades (well differentiated *n* = 2, moderately differentiated *n* = 6, and poorly differentiated *n* = 2) and normal colonic epithelia (*n* = 2). The expression of serglycin was diffuse, granular, and almost exclusively cytoplasmic in all the colon cancer cases, as well as in normal colon epithelia (Figure 2). Notably, grade 2 and 3 neoplasms displayed very strong serglycin immunoreactivity (Figures 2(a), 2(c) and 2(d)), whereas the intensity of the two grade 1 malignancies was weak (+1) and moderate (+2) (Figure 2(b)). The case that exhibited weak immunopositivity was a low-grade adenocarcinoma, originating from a villous adenoma (Figure 2(b)). A careful examination of the samples revealed that, in all malignancies lymphocytes, plasma cells, stromal and endothelial cells were immunopositive for serglycin (Figure 2(d)). Interestingly, the intensity of serglycin immunoexpression was augmented at the invasive front of the carcinomas (Figure 2(d)). Normal large intestine epithelium displayed moderate serglycin immunoreactivity, whereas serglycin staining was weak in plasma cells, lymphocytes, and stromal cells of the lamina propria (Figure 2(e)). Smooth muscle cell layer of colon also displayed elevated serglycin immunoreactivity (Figure 2(e)).

#### 3.3.2. Breast Cancer

**Figure 3:**
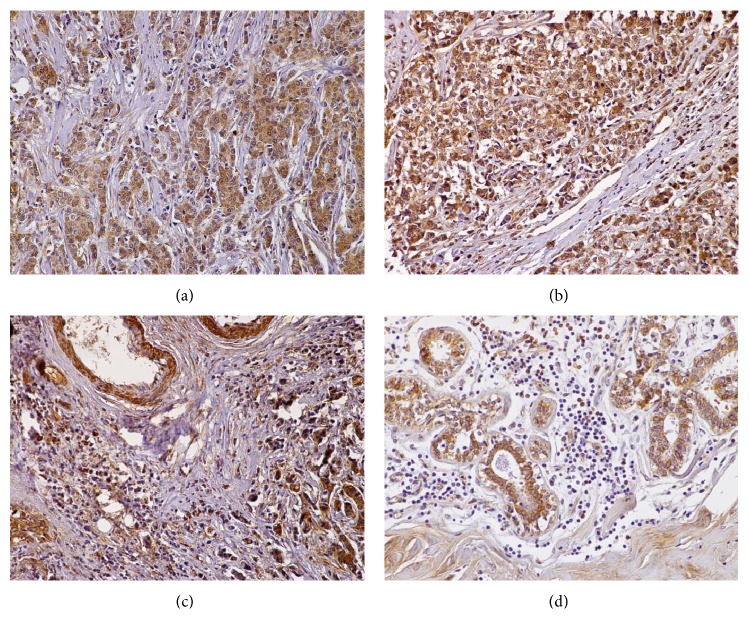
(a) Grade 2 and (b) grade 3 breast ductal carcinomas displaying strong granular, cytoplasmic serglycin immunoreactivity. (c) Grade 3 infiltrating breast carcinoma. Note that the tumor cells as well as chronic inflammatory and stroma cells show strong serglycin expression. (d) Section from a benign breast lesion showing that serglycin is expressed in the glands and the stroma. However, inflammatory cells display minimal serglycin positivity. Original magnifications 20x.

The tissue microarray also contained 7 grade 2 and 3 grade 3 breast carcinomas, as well as 2 cores obtained from 2 different normal breasts. All the neoplasms and the normal glands displayed diffuse, strong (3+), cytoplasmic, granular serglycin immunoreactivity (Figures 3(a)–3(d)). The vast majority of plasma cells, lymphocytes, and endothelial and stromal cells were also serglycin positive. Of note, the expression of serglycin levels was enhanced in the plasma cells, lymphocytes, and stromal cells from grade 3 tumors (Figure 3(c)). The expression of serglycin in the lymphocytes and plasma cells of normal tissues was minimal (Figure 3(d)).

#### 3.3.3. Prostate Cancer

**Figure 4:**
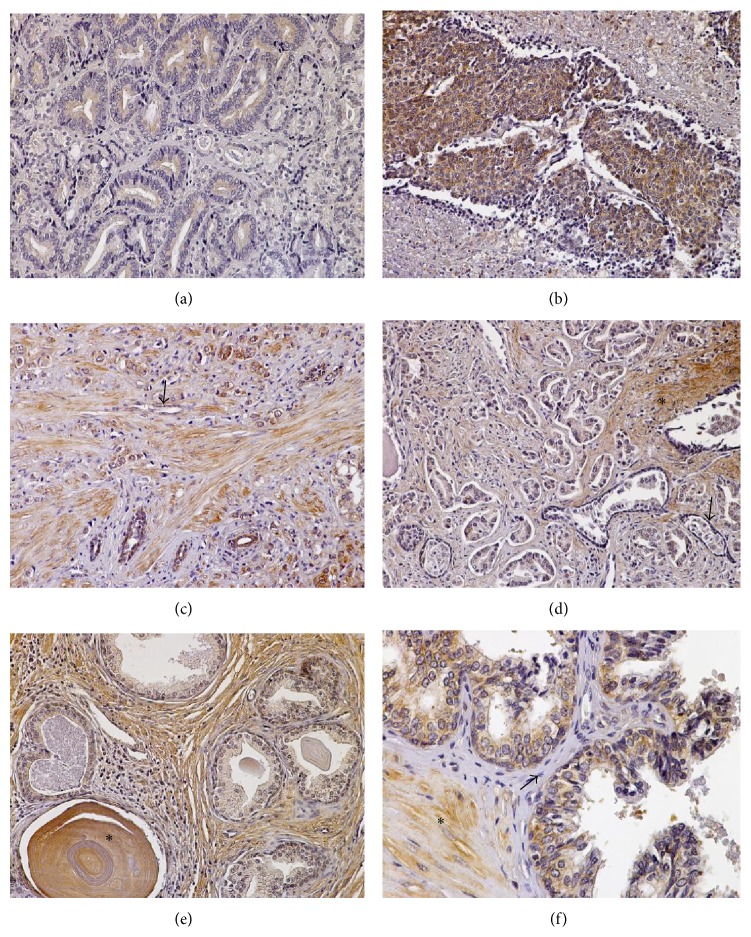
(a) A low-grade prostate adenocarcinoma showing weak serglycin expression. (b) A high-grade prostate adenocarcinoma with strong cytoplasmic, diffuse immunoreactivity for serglycin. (c) Note that tumor, endothelial (arrow), and stroma cells are positive for serglycin in this high-grade prostate adenocarcinoma. (d) In this case of low-grade prostatic adenocarcinoma, malignant and benign prostate glands, as well as fibromuscular stroma (asterisk), display serglycin immunopositivity; on the contrary, basal cells of the benign prostate glands (arrow) are negative for this protein. (e) Benign prostate hyperplasia. Benign glands, stromal cells, chronic inflammation cells (plasma cells and lymphocytes), and corpora amylacea (asterisk) are serglycin-reactive. Also note that basal cells do not exhibit serglycin immunoreactivity. (f) In this section of normal prostate, epithelial cells and smooth muscle (asterisk) are positive, whereas fibrovascular core cells (arrow) are negative for serglycin. (a–e) Original magnification 20x; (f) original magnification 40x.

Two low-grade (Gleason score 4), 3 moderate-grade (Gleason score 6), and 5 high-grade prostate adenocarcinomas (Gleason score 10) were examined for serglycin expression. Serglycin was detected in both the neoplastic and the normal prostatic epithelia. Its immunoexpression was cytoplasmic, granular, and diffuse in all the tumors examined (Figures 4(a)–4(d)). Interestingly, none of the low-/moderate-grade tumors displayed high (3+) serglycin cellular levels. On the contrary, there was no high-grade adenocarcinoma exhibiting low-grade (1+) serglycin immunoreactivity in the samples tested. Endothelial cells in tumor stroma had elevated serglycin cellular levels (Figure 4(c)). Low levels of serglycin were also detected in the cytoplasm of hyperplastic and normal prostate glands (Figures 4(e) and 4(f)). Notably, basal cells of prostatic glands and fibrovascular cores were negative (Figure 4(f)), while smooth muscle and corpora amylacea were positive for this proteoglycan (Figures 4(e) and 4(f)).

#### 3.3.4. Lung Cancer

**Figure 5:**
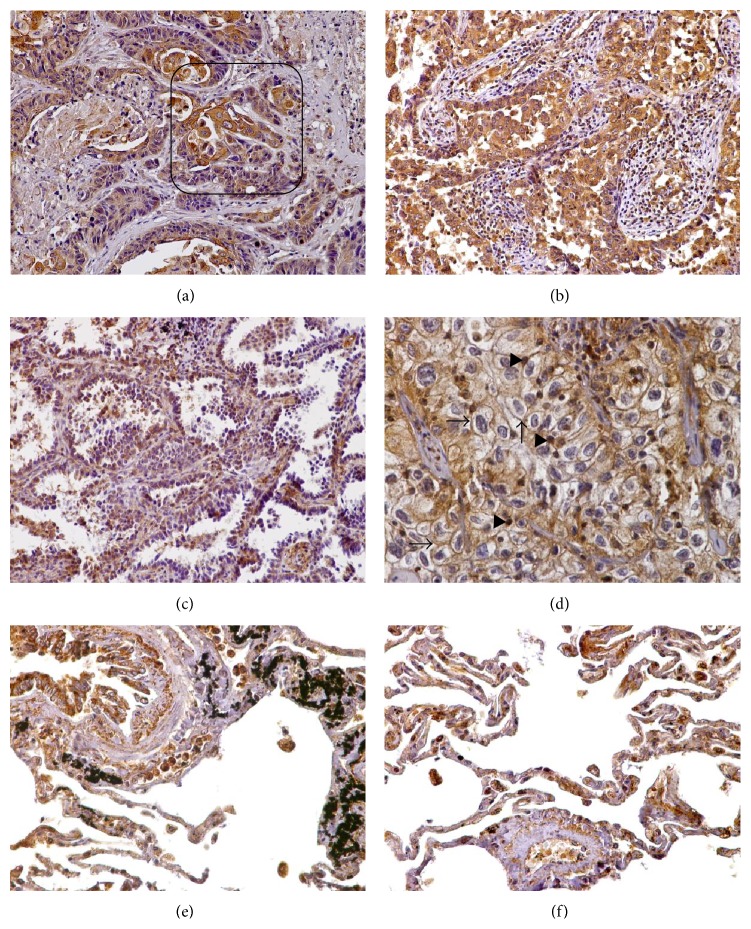
(a) A moderately differentiated squamous cell lung carcinoma displaying strong cytoplasmic serglycin immunoreactivity. Note that stroma and chronic inflammation cells are also intensively positive for serglycin, primarily at the invasive fronts (box). (b) A moderately differentiated lung adenocarcinoma that shows strong serglycin immunoexpression. (c) Serglycin immunopositivity in a low-grade bronchoalveolar adenocarcinoma. (d) Note that in this case of large cell undifferentiated lung carcinoma serglycin mainly displays cytoplasmic, granular immunoreactivity; however, it also displays nuclear (arrowheads) and the cell membranous (arrows) localization. (e and f) In normal lung sections, serglycin is detected in the epithelium of bronchioles as well as in alveolar macrophages and pneumocytes. Original magnifications 20x.

We also examined the distribution of serglycin in 5 squamous cell carcinomas (moderately and poorly differentiated) and 5 adenocarcinomas (moderately and poorly differentiated), including a large cell carcinoma and a bronchoalveolar carcinoma. The intensity of serglycin immunoreactivity was strong (+3) and its distribution was diffuse, cytoplasmic, and granular in all the lung carcinomas examined (Figures 5(a)–5(d)). Endothelial cells, plasma cells, and lymphocytes displayed intense serglycin immunoreactivity. Stromal fibroblasts were also serglycin positive, primarily at the invasive fronts (Figure 5(a)). Notably, in the one case of large cell carcinoma that was involved in the tissue microarray, the tumor cells displayed strong membrane immunoreactivity as well as nuclear staining (Figure 5(d)). As regards normal lung, serglycin was strongly expressed in macrophages, pneumocytes, and bronchial epithelium cells (Figures 5(e) and 5(f)).

## 4. Discussion

Although numerous studies have shown that serglycin is involved in hematological malignancies, not much information has been published on the expression and distribution of this proteoglycan in solid tumors [1, 2, 23]. Only few studies have shown that serglycin augmented expression is associated with cancer cell aggressiveness and disease progression in hepatocellular [8], breast [9], and nasopharyngeal cancer [10].

The results of the present pilot study reveal that serglycin is highly expressed and secreted by more aggressive cancer cells. Serglycin is secreted in elevated levels in cancer cells mutated in KRAS or HER1/EGFR, the fact that may suggest an implication of EGFR-RAS pathway in the biosynthesis and more importantly in secretion of serglycin. As shown in previous studies, the overexpression of serglycin is correlated with the establishment of more aggressive mesenchymal cancer cell phenotype and promotes cancer cells proliferation, especially in nonadhesive matrices, migration, and invasion both* in vitro* and* in vivo* [9, 10]. All cancer cells analyzed till now synthesize serglycin, which is modified with CS chains and not heparin as in mast cells [3, 4]. The glycanation of serglycin may modulate the biological functions of tumor-derived serglycin since it seems to be important for serglycin's biological functions [23]. For example, serglycin modified with CS chains enriched with 4-sulfated disaccharides is responsible for conferring tumor cells resistance against immune system attack mediated by the activation of the classical and lectin pathways of complement [9, 12]. Furthermore, the establishment of aggressive breast cancer phenotype following upregulation of NEDD9 is accompanied by increased expression of serglycin, its cell surface binding partner CD44, and CS synthesizing enzymes [24]. This study has demonstrated that serglycin and CD44 carry CS modified with disulfated disaccharides being sulfated at C4 and C6 of* N*-acetyl-galactosamine (CS-E units). It has been shown that CS-E plays a key role in promoting and regulating breast cancer progression and metastasis and possibly stem cell phenotype [24]. CS chains of serglycin are mainly responsible for binding to CD44 and collagen type I. Serglycin binding to CD44 most likely mediates the cell surface localization of serglycin [5, 7] that further interacts with matrix collagen type I thus promoting the adhesion of tumor cells [5, 11]. The core protein of serglycin is involved in the interaction with MMP-13 [25] and proMMP-9 and the formation of complexes [26, 27]. Both hemopexin-like domain and the fibronectin-like module of proMMP-9 are implicated in the interaction with serglycin. The formation of complexes modulates the mode of activation of the enzyme and its interaction with substrates [26, 28].

In our study, the expression of a splice variant of serglycin that lacks exon 2 was detected although the mature protein encoded by this splice variant was not detected in our western blots likely due to its minor expression. The alternative splicing that occurs from the loss of exon 2 has been previously demonstrated in neutrophils and has been associated with the presence of a unique DNase I-hypersensitive site in exon 2 in these cells [22]. The biological importance of exon 2 deletion for serglycin is unclear. Nevertheless, it may participate in several serglycin functions since the N-terminal portion of the sequence encoded by exon 2 contains a potential heparin binding site YPTQRARYQWVRCNP and the possible dimerization of the peptide sequence via the cysteine residue results in significant binding affinity to low molecular weight heparin [29]. It has been proposed that heparin binding site and dimerization of serglycin core together with glycosaminoglycan chains regulate the binding of serglycin with other molecules [23, 29].

The biosynthesis of serglycin by normal and cancer cells themselves was detected in cancer tissues by immunohistochemistry. Serglycin was found to be expressed in normal epithelial cells exhibiting cytoplasmic staining. The biological function of serglycin in normal epithelium is still unknown. Further studies are needed to elucidate whether serglycin is located within vesicles in epithelial cells, is either secreted constitutively or upon stimulation, or remains exclusively intracellularly. Although a limited number of cases were examined, our data have shown a strong diffuse cytoplasmic distribution for serglycin in more aggressive cancer cells in most cases. The pattern of cytoplasmic staining was granular suggesting the packaging of serglycin in secretory granules within cancer cells. It is known that serglycin is localized in secretory granules in hematopoietic cells as well, where it plays a role in the packaging and secretion of many molecules [23]. A similar function of serglycin in cancer cells cannot be excluded.

In this pilot study, serglycin was also found to be expressed in higher levels in stromal cells in tumor stroma as compared to normal tissue. Serglycin was expressed by inflammatory and endothelial cells and fibroblasts in reactive tumor stroma especially at the invasive fronts suggesting a crucial role for serglycin in cancer spread. It has been shown that serglycin expression is markedly upregulated in cancer-activated fibroblasts among other inflammatory mediators and a disintegrin and metalloproteinase with thrombospondin motifs 1 (ADAMTS-1) promoting cancer cell invasion [30]. Serglycin is also implicated in the biosynthesis and secretion of growth factors and inflammatory mediators such as HGF and CXCL1 in plasma [5] and endothelial cells [31, 32], respectively, as well as in the production of proteases in several inflammatory cells, which have proven roles in cancer cell growth and spread [3, 23]. Recently, it has been shown that serglycin is highly expressed in tumors in RIP1-Tag2 mouse model for spontaneous insulinoma formation [33]. It is suggested that the majority of serglycin comes from stromal cells. When RIP1-Tag2 mouse model is crossed into serglycin deficient mice, a decrease only in the volume of developed tumors but not in their number is noticed [33]. Furthermore, the frequency of angiogenic islets is decreased in serglycin deficient RIP1-Tag2 mice and this is also accompanied with reduced biosynthesis of proangiogenic modulators such as vascular endothelial growth factor and HGF [33]. The absence of serglycin also enhances the functionality of tumor vessels, which were better perfused than that developed in tumors in serglycin wild type mice [33]. These findings suggest the involvement of serglycin in the development of a proangiogenic environment.

The overexpression of serglycin by cancer and stromal cells may augment the expression of inflammatory mediators, growth factors, and proteolytic enzymes. These factors may act in autocrine and/or paracrine manner affecting the behavior of both stromal and cancer cells. It is likely that serglycin is implicated in the establishment of a flourishing inflammatory tumor microenvironment that drives cancer cell growth and spreading.
